# Healthful Food Availability in Stores and Restaurants — American Samoa, 2014

**Published:** 2015-03-20

**Authors:** Seung Hee Lee-Kwan, Gayathri Kumar, Patrick Ayscue, Marjorie Santos, Lisa C. McGuire, Heidi M. Blanck, Motusa Tuileama Nua

**Affiliations:** 1 Epidemic Intelligence Service, CDC; 2 Division of Diabetes Translation, National Center for Chronic Disease Prevention and Health Promotion, CDC; 3 Division of Nutrition, Physical Activity, and Obesity, National Center for Chronic Disease Prevention and Health Promotion, CDC; 4 American Samoa Department of Health

American Samoa, one of the U.S.-affiliated Pacific Islands, has documented the highest prevalence of adults with obesity (75%) in the world (1). The nutritionally poor food and beverage environment of food retail venues has been suspected to be a contributing factor (2), although an evaluation of these venues in American Samoa has not been conducted. In January 2014, American Samoa established an Obesity Task Force to develop policies and strategies to combat obesity. To inform the efforts of the task force, the American Samoa Department of Health and CDC conducted a baseline assessment of the availability, pricing, and promotion of healthful foods at retail food venues. Previously validated food environment assessment tools were modified to incorporate American Samoa foods and administered in a geographically representative sample of 70 stores (nine grocery stores and 61 convenience stores) and 20 restaurants. In convenience stores, healthful items were not found as available as less healthful counterparts, and some healthful items were more expensive than their less healthful counterparts. For restaurants, 70% offered at least one healthful entrée, whereas only 30% had healthful side dishes, such as vegetables. Actions to promote healthy eating, such as providing calorie information, were rare among restaurants. Improving availability, affordability, and the promotion of healthful foods in American Samoa stores and restaurants could support healthy eating among American Samoa residents.

American Samoa consists of five islands and two coral atolls with 54,517 residents in 2014; most residents are Native Hawaiian or other Pacific Islander (91.6%) (3). American Samoa and other Pacific Islands have unique food environments that rely heavily on imported foods (4). On average, freighters carrying imported foods arrive in American Samoa approximately twice a month (Personal communication, Va’a Tofaeono, Program Coordinator, American Samoa Department of Health). Given that most imported foods are transported by sea, they are often processed to prolong their shelf lives. In addition, the variety of products that are imported is limited (4).

To identify retail food sources, a pair of two-person teams canvassed Tutuila Island by car and foot, visually inspecting and identifying the location and business status (i.e., in-business, out-of-business, or under renovation) of all retail food vendors. Tutuila Island was selected for data collection because it is the home of over 95% of the American Samoa population. North American Industry Classification System (NAICS) code definitions were used to define grocery stores, convenience stores, and restaurants.* Stores with more than two cash registers were classified as grocery stores, and stores with only one cash register were classified as convenience stores (5). A total of 12 grocery stores, 110 convenience stores, and 48 restaurants were identified. A geographically representative sample of 70 stores (nine grocery stores and 61 convenience stores) and 20 restaurants were selected for assessment.

Previously validated nutrition environment survey instruments for stores (Nutrition Environment Measures Surveys-Stores or NEMS-S) and restaurants (Nutrition Environment Measures Surveys-Restaurants or NEMS-R)† were modified to reflect foods available in American Samoa to assess the availability, pricing, and promotion of more healthful foods. Descriptive statistics were used to estimate the frequency of product availability, pricing, and promotion information for healthful and less healthful foods items in stores and restaurants. More healthful food items were defined according to NEMS-S and NEMS-R criteria, augmented by the Dietary Guidelines for Americans for 2010.§ Food items were classified as healthful without a corresponding less healthful counterpart (e.g., fresh fruits, fresh vegetables, frozen fruits, frozen vegetables, canned fruits, canned vegetables, and canned beans) or more healthful food items that have a corresponding less healthful counterpart (e.g., lean meats [≤10% fat ground beef], low sugar cereals [<7g sugars per serving], whole grain breads, reduced-fat dairy products, and canned soup [≤100 kcal per serving]). More healthful beverages were defined as low-fat or skim milk, 100% fruit juice, zero-calorie sodas, and bottled water.

Findings showed that fresh fruits and vegetables were available more often in grocery stores than convenience stores but prices were comparable (e.g., availability of Chinese cabbage was 100% and the average price was $1.32/lb [$2.91/kg] in grocery stores versus 38% and an average price of $1.38/lb [$3.04/kg] in convenience stores) (Table 1). In convenience stores, more healthful items were not available as often as corresponding less healthful items (e.g., lean ground beef was available in no stores versus regular ground beef available in 45 [74%] stores). Some more healthful items were more expensive on average than their corresponding less healthful counterparts (e.g., $1.88 for 100% whole-wheat bread versus $1.12 white bread) (Table 2).

Among restaurants, 14 (70%) offered at least one healthful entrée but only six (30%) had healthful side dishes such as vegetables. When available, healthful entrées were, on average, 9% more expensive than less healthful entrées ($7.47 versus $6.83; [n = 14]) and healthful sides were, on average 15% more expensive than less healthful sides ($4.51 versus $3.91; [n = 6]). Educational or promotional factors to encourage healthful eating, such as calorie counts, other nutrition information, or menu notations encouraging healthy substitutions, were available in approximately 5% of surveyed restaurants.

## Discussion

Overall, findings in the American Samoa stores were comparable with those from studies conducted in stores in the continental United States (including in urban and rural environments), showing that more healthful items were limited in convenience stores. For example, an assessment conducted in low-income urban neighborhoods of Philadelphia in 2011 (6) found that just over half of surveyed convenience stores sold whole grain bread (56%); similarly, low availability of whole grain breads was found in American Samoa (39%). In American Samoa, the average prices of the commonly available fruits and vegetables were comparable in grocery and convenience stores. In the continental United States, fresh fruits and vegetables were often more expensive in convenience stores than grocery stores (7). Possible reasons for this discrepancy are that most American Samoan convenience stores had a refrigeration system and some fruits and vegetables were obtained locally (e.g., Chinese long beans and cabbage). A refrigeration system extends the shelf-life of certain types of fresh fruits and vegetables, which can impact variety, quality, waste, and the pricing of products. Many convenience store managers in the continental United States perceive lack of refrigeration options in smaller stores as a barrier to selling more healthful foods (8).

The restaurant findings in this assessment were similar to other studies conducted in the continental United States that showed that more healthful items were less available and promotions were limited (9), indicating opportunities to improve healthful item availability and promotion in American Samoa. For example, an assessment conducted in a rural community in Minnesota in 2011 found that 4% of restaurants highlighted healthy options, similar to restaurants in American Samoa (5%) (10). Using these findings, the American Samoa Department of Health initiated conversations with the owners of restaurants that offer more healthful items and is identifying ways to promote those restaurants to consumers.

The findings in this report are subject to at least four limitations. First, although the sample was geographically representative, it was not a random sample because stores in remote villages were included at the request of the American Samoa Department of Health. Second, this assessment covered only Tutuila Island, excluding the other four islands and two atolls; however, Tutuila Island accounts for >95% of the American Samoa population. Third, although the assessment tool covered many of the more healthful items available in stores, some items, such as dried legumes and brown rice, were not included. Finally certain types of healthful traditional foods such as papayas and bananas were often sold by roadside vendors but not included in this assessment.

What is already known on this topic?

American Samoa has the highest prevalence of obesity (75%) globally. A nutritionally poor food and beverage environment of food retail venues has been suspected to be a community-level predictor of obesity.

What is added by this report?

In April 2014, separate store and restaurant Nutrition Environment Measurement Surveys found that healthful foods in American Samoan stores and restaurants were infrequently available, often cost more, and were promoted less.

What are the implications for public health practice?

Potential action items include reviewing existing policies that facilitate or pose barriers to healthful food distribution to American Samoa stores and restaurants and encouraging stores and restaurants to incorporate affordable local ingredients such as locally grown vegetables to avoid high shipping costs.

CDC is collaborating with the American Samoa Department of Health to develop a comprehensive report that will summarize the findings to inform the task force’s efforts. Potential action items include reviewing existing policies that facilitate or pose barriers to more healthful food distribution and establishing commitments from vendors to incorporate more affordable local ingredients, such as locally grown vegetables, to avoid high shipping costs.

## Figures and Tables

**TABLE 1:** Availability and mean prices of fruits and vegetables in nine grocery stores* and 61 convenience stores† — American Samoa, April 2014

Type of food (unit price)	Availability		Price (U.S. $)	
	
	Grocery stores	Convenience stores	Grocery stores	Convenience stores
			
	%	%	Mean	Mean
**Any fresh fruit**	100	74	—	—
**Top-selling fruits**				
Oranges (1lb [454 g])	100	70	1.53	1.56
Apples (1lb [454 g])	89	52	1.60	1.73
**Any fresh vegetables**	100	93	—	—
**Top-selling vegetables**				
Carrots (1lb [454 g])	100	72	1.04	1.25
Chinese cabbage (bag§)	100	38	1.32	1.38
Chinese long beans (bag§)	89	49	1.40	1.39

* Stores with more than one cash register.

† Stores with one cash register.

§ Each bag weighed approximately 1 lb (454 g).

**TABLE 2:** Availability and mean prices of selected more healthful and less healthful food items in 61 convenience stores — American Samoa, April 2014

Type of food (unit price)	Availability		Price (U.S. $)	
	
	Less healthful	More healthful	Less healthful	More healthful
			
	%	%	Mean	Mean
**Meats and cheese**				
Ground beef (1lb [454 g])	74	0	3.14	—
Canned meats (7 oz [198 g])	98	98	2.39	1.82
Hot dogs (12 oz [340 g])	69	89	1.22	1.29
Cheese (10 oz [283 g])	79	0	1.97	—
**Beverages**				
Soda (12 oz [355 mL])	98	74	0.75	0.75
Juice (16 oz [473 mL])	93	66	1.15	1.44
Bottled water (17 oz [500ml])	0	98	—	1.00
**Bread and cereal**				
Bread (1lb [454 g])	80	39	1.12	1.88
Cereal (12 oz [340 g])	95	62	4.49	4.65

## Footnotes

* Available at http://www.census.gov/mrts/www/naicsdef.html.

† Available at http://www.med.upenn.edu/nems/measures.shtml.

§ Available at http://www.health.gov/dietaryguidelines/2010.asp.
